# Influenza and Respiratory Syncytial viral infections in Malaysia: Demographic and Clinical perspective

**DOI:** 10.12669/pjms.301.4272

**Published:** 2014

**Authors:** M.M. Rahman, K.K. Wong, A. Hanafiah, I. Isahak

**Affiliations:** 1 M.M. Rahman, Department of Medical Microbiology & Immunology, University Kebangsaan Malaysia Medical Centre, Kuala Lumpur, Malaysia.; 2 K.K. Wong, Department of Medical Microbiology & Immunology, University Kebangsaan Malaysia Medical Centre, Kuala Lumpur, Malaysia.; 3 A. Hanafiah, Department of Medical Microbiology & Immunology, University Kebangsaan Malaysia Medical Centre, Kuala Lumpur, Malaysia.; 4 I. Isahak, Department of Medical Sciences,Faculty of Medicine & Health Sciences, University Sains Islam, Malaysia.

**Keywords:** Influenza virus, Respiratory syncytial virus, Prevalence, Demography, Real-time reverse transcriptase-PCR, Immuno Fluorescence Technique

## Abstract

***Objective:*** Respiratory infections represent a major public health problem worldwide. The study aimed to determine the prevalence of respiratory syncytial and influenza virus infections and analyzed in respect to demography and clinical perspective.

***Methods*** **:** The specimens were processed by cell culture and immunofluorescent assay (IFA) and real-time reverse transcriptase-PCR (rRT-PCR) for detection of respiratory viruses.

***Results*** **:** Out of 505 specimens 189 (37.8%) were positive, in which RSV was positive in 124(24.8%) cases and influenza A was positive in 65(13%) cases. Positive cases for influenza virus A and RSV were analyzed based on demography: age, gender, ethnicity and clinical symptoms. There were no significant differences among gender, ethnicity and clinical symptoms in both RSV and influenza A virus infections. It was observed that children below 3 years of ages were more prone to RSV infections. On the contrary, influenza virus A infected all age groups of humans.

***Conclusion:*** RSV infects mostly child below 3 years of age and influenza virus infects all age group. No specificity of RSV and influenza infection in relation to demography.

## INTRODUCTION

Respiratory viruses are the major causes of respiratory illness throughout the world.^1^ Among these viruses influenza and respiratory syncytial virus (RSV) play predominant role.^2^ These viruses cause morbidity and mortality of young children, elderly and immune-compromised patients. The most common clinical manifestations of these viral infections range from fever, sore throat, and myalgia to more serious complications like bronchitis, pneumonia and death.^3^

Three known types of influenza viruses (A, B, and C) currently circulate in the human population, types A and B associated with clinically important respiratory illness.^4^ Respiratory syncytial virus (RSV) is the best known for its tendency to cause bronchiolitis in infants, nevertheless it can infect all age groups causing upper and lower respiratory tract infections ranging in severity from subclinical infections to pneumonia and death.^5^

Each year, influenza viruses cause illness millions of cases associated with various respiratory syndromes and approximately 500,000 deaths.^6^ Globally, about 20% of children and 5% of adults develop symptomatic influenza each year.^7^ Similarly RSV is associated with 40%–90% of bronchiolitis cases in children less than 5 years of age and 50% of pneumonia cases in children less than 2 years of age.^8^ Therefore, respiratory virus infections represent a major public health problem because of their worldwide occurrence, ease of spread in the community and considerable morbidity and mortality. New respiratory viruses with epidemic and pandemic potential continue due to their genomic nature.^9^

In Malaysia, the Institute for Medical Research (IMR), Kuala Lumpur screened respiratory illness on 7,117 respiratory specimens during 2005-2009 and reported for the identification of influenza viruses 17.3% in 2005, 31.6% in 2006, 12.8% in 2007, 10.2% in 2008 and 13.5% in 2009.^10^

Appropriate diagnosis of viral agent provides guidance for the prompt management of the patients showing respiratory illness. There is limited data available on the study of respiratory viruses of Malaysian patients. Detailed study in relation to respiratory viruses especially respiratory syncytial and influenza viruses considering patients demography has not been carried out before.

## METHODS

***Study population:*** The study was undertaken at University Kebangsaan Malaysia Medical Centre (UKMMC) from March 2011 to August 2011. 505 throat swabs and respiratory aspirates were collected during the period. These were sent to the laboratory of Medical Microbiology and Immunology for virus isolation and identification.

***Case definition based on clinical symptoms:*** Mild: Fever and cough and influenza-like illness (ILI) were considered mild clinical symptoms. Moderate: Different types of pneumonia were considered moderate clinical symptoms. Severe: Acute bronchiolitis, acute exacerbation of bronchial asthma, chronic lung disease and chronic obstructive airway disease were considered severe clinical symptoms.

***Demography of the patients:*** Patients of all ages, gender and ethnicity and the nature of respiratory illness were recorded from the patients’ information sheet provided with the specimens by the clinicians.

***Ethical approval:*** The study protocol was approved by UKMMC Ethical Committee (FF-320-2011) 

***Propagation of viruses in cell culture:*** Madin-Darby Canine Kidney (MDCK) cells (ATCC number, CCL-34^TM^) an HEp-2 cells (ATCC number, CCL-23^TM^) were purchased from ATCC (Manassas, VA 20110, USA.) and used for the propagation and initial detection of viruses based on cytopathic effect. Method of Chew et al.(2012)^11^ was followed for the whole process with slight modifications.

***Indirect Immunoflourescence Assay:*** The presence of a specific virus was confirmed by Indirect Immunoflourescence staining. The Light Diagnostics™ Respiratory Panel 1 Viral Screening and Identification Kit (Millipore, USA) were used for the qualitative confirmation of influenza A, influenza B, respiratory syncytial virus viruses. Method described by Chew et al.(2012)^11^ was also followed in this regard.

***Molecular detection of influenza virus and RSV*** **: **The specific nucleotide for the primer and probe in the gene coding from the complete genome of RSV and Influenza viruses were obtained from gene bank. Molecular assay was carried out for the identification of viruses as per the procedure of Ken et al.(2012).^12^

***Statistical analysis***: Analysis was carried out by using the Statistical Package for Social Sciences SPSS version 15.0 (SPSS Inc., Chicago, USA). The Chi-square test was used to analyze categorical variable where *P*< 0.05 was considered statistically significant.

## RESULTS

***Total number of Influenza and RSV*** *:* Out of 505 specimens tested, 124 (24.6%) and 65 (12.9%) were positive for RSV and influenza A virus, respectively. No influenza B was detected. The average isolation rate per month through March to August 2011 was 19.42% for RSV and 9.50% for influenza A. The highest number of positive cases of both RSV and influenza were identified in June.

**Fig.1:**
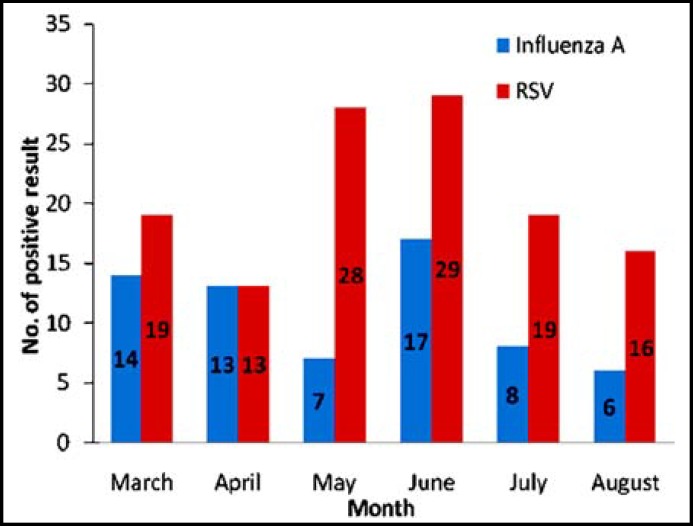
Positive for RSV and influenza A viruses identified at UKMMC through March 2011 to August 2011

***Demographic features of RSV and Influenza virus infected patients:*** Demographic and clinical symptoms of the patients those were positive for respiratory syncytial and influenza viral infections through March 2011 to August 2011 have been presented in Table I and II, respectively.

**Table-I:** Demographic and clinical symptoms of RSV positive patients

*Parameter*	*Variable*	*No. (%) patients* *(n=124)*	*P value*
Gender	Male	74 (59.68%)	0.985
	Female	50 (40.32%)	
Ethnic	Malay	87 (70.16%)	
	Chinese	11 (8.87%)	0.461
	Indian and others	26 (20.97%)	
Age	<3 years old	104 (83.87%)	
	3-10 years old	13 (10.48%)	0.000
	>10 years old	7 (5.65%)	
Type of specimens	Throat swab	78 (62.90%)	0.737
	Nasopharyngeal aspirate (NPA)	46 (37.10%)	
Clinical symptoms	Mild	39 (31.45%)	0.572
	Moderate	64 (51.61%)	
	Severe	21 (16.94%)	

No significant differences were observed among patients’ gender race, type of specimens and clinical symptoms with RSV infections (Table 1). However, statistically significant difference was observed between RSV infections and the age group (*P* < 0.0001).

It reveals that out of 124 RSV positive patients 59.68% and 40.32% were male and female, respectively. In relation to ethnic group Malay 70.16%, Chinese 8.87% and Indian and others were 20.97%. Considering age, less than 3 years old patients 83.87%, 3-10 years old 10.48% and more than 10 years old patients were 5.65%. Throat swabs specimens 62.90% and Nasopharyngeal aspirates were 37.10%. Patients of mild respiratory symptoms 31.45%, moderate 51.61% and severe respiratory symptoms were 16.94%.

**Table-II:** Demographic and clinical symptoms of the influenza A virus positive patients

*Parameters*	*Variable*	*No. (%) patients* *(n=65)*	*P value*
Gender	Male	35 (53.85%)	0.311
	Female	30 (46.15%)	
Ethnic	Malay	50 (76.92%)	0.121
	Chinese	5 (7.69%)	
	Indian and others	10 (15.38%)	
Age	<3 years old	44 (67.69%)	0.908
	3-10 years old	13 (20.00%)	
	>10 years old	8 (12.31%)	
Type of specimens	Throat swab	41 (63.08%)	0.846
	Nasopharyngeal aspirate (NPA)	24 (36.92%)	
Clinical symptoms	Mild	21 (32.31%)	0.485
	Moderate	35 (53.85%)	
	Severe	9 (13.84%)	

In influenza A infected patients, no statistically significant difference was observed for all the parameters (Chi-square test, *P>*0.05)

It reveals that out of 65 influenza A virus positive patients 53.85% and 46.15% were male and female, respectively. In relation to ethnic group Malay 76.92%, Chinese 7.69% and Indian and others were 15.38%. Considering age, less than 3 years old patients 67.69%, 3-10 years old 20% and more than 10 years old patients were 12.31%. Throat swabs specimens 63.08% and Nasopharyngeal aspirates were 36.92%. Patients of mild respiratory symptoms 32.31%, moderate 53.58% and severe respiratory symptoms were 16.94%.

## DISCUSSION

In the present study it was observed that of the 505 specimens analysed by cell culture, indirect immunoflurecnt assay and real-time reverse transcriptase-PCR (rRT-PCR) in which 124 (24.6%) and 65 (12.9%) were positive for RSV and influenza A virus, respectively. In Malaysia published data related to present study is scanty. Therefore, we are unable to compare the results with local data. However, the Institute for Medical Research (IMR), Kuala Lumpur screened respiratory illness of 7,117 respiratory specimens during 2005-2009 and reported for the identification of influenza viruses 17.3% in 2005, 31.6% in 2006, 12.8% in 2007, 10.2% in 2008 and 13.5% in 2009.^10^ In the report they did not mention RSV infections.

In the study 124(24.6%) patients were positive for RSV infections in Malaysia. In positive patients 59.68% and 40.32% were male and female, respectively. In relation to ethnic group Malay 70.16%, Chinese 8.87% and Indian and others were 20.97%. Considering age, less than 3 years old patients 83.87%, 3-10 years old 10.48% and more than 10 years old patients were 5.65%. Throat swabs specimens 62.90% and Nasopharyngeal aspirates were 37.10%. Patients of mild respiratory symptoms 31.45%, moderate 51.61% and severe respiratory symptoms were 16.94%. It indicates that age below 3 years is the highest infection of RSV infection in Malaysia.

On the contrary, it was observed that 12.9% patients were positive for influenza A virus in which 53.85% and 46.15% were male and female, respectively. In relation to ethnic group Malay 76.92%, Chinese 7.69% and Indian and others were 15.38%. Considering age, less than 3 years old patients 67.69%, 3-10 years old 20% and more than 10 years old patients were 12.31%. Throat swabs specimens 63.08% and nasopharyngeal aspirates were 36.92%. Patients of mild respiratory symptoms 32.31%, moderate 53.58% and severe respiratory symptoms were 16.94%.

In Malaysia the total population has been divided into 65% Malays, 14% Chinese and 21% Indian and others. In our study majority of the positive patients were mainly Malays which reflect the normal distribution of the total population of the country. 

Gender wise, male makes up 62% and female is 38% of the total specimens collected from the patients. Previous research suggests a higher percentage of respiratory infections in women (9.0%) compared to men (7.1%).^13^ Other authors reported that women might be more exposed to airborne infections because of their role in child care and more contacts with older people who live in the family or with other close relatives.^14^ However, our data did not reflect the observations mentioned by the above authors because of 46.15% of the patients in this study were female.

An urban community based surveillance report in Bangladesh showed that the incidence of influenza among children aged less than 5 years with acute respiratory infection was 10 per 100 person yearly.^15^ The incidence of respiratory illness (10 per 100 person years in 2008) was also similar to that observed in rural India in children aged less than 3 years with acute respiratory infection and influenza (14 per 100 person yearly).^16^ Another study showed that children aged <11 years suffered a high burden of influenza virus infection.^17^ It is probably due to predilection of the virus to the young make them more vulnerable to pick up infection.

In case definition we classified the patients in three categories based on clinical symptoms. In our study most of the patients were under moderate clinical symptoms in case of both RSV and influenza A infections.

A study conducted in USA estimated that 2.1 million children under the age of five required medical attention each year due to RSV.^18^ The authors further mentioned that 61% of the patients were children of 2–5 years of age. This showed that impact of RSV is greater during early age of children. Another study estimated that, globally, RSV caused almost 34 million cases of respiratory illness in children less than 5 years of age, 10% of them requiring hospitalization.^19^ Similar results cited by other authors and mentioned that there were no significant differences in terms of gender and ethnicity between patients infected with different viruses.^20^ It was observed that clinical symptoms caused by different respiratory viruses were similar. Statistical analysis showed no relationships between genotypes of influenza A with clinical symptoms. A study showed that signs and symptoms of influenza caused by pandemic H1N1 influenza A virus were similar to those of seasonal influenza, although gastrointestinal manifestations appear to be more common with pandemic H1N1 influenza.^21^ Another study compared adult’s infection with pandemic H1N1-2009 to those with H3N2 found no statistically significant differences of symptoms.^22^ In our study no statistically significant difference was observed in different demographic parameters in influenza A virus infection. Published data on demography in relation to influenza A infection is scanty to compare.

In conclusion, the study highlights that RSV and influenza A are the most prevalent respiratory viruses in Malaysia. RSV infects mostly child below 3 years of age and influenza virus infects all age group. Both RSV and influenza A infection did not show any relationship with patients’ demography.
